# Intestinal microbiome and NAFLD: molecular insights and therapeutic perspectives

**DOI:** 10.1007/s00535-019-01649-8

**Published:** 2019-12-16

**Authors:** Haiming Hu, Aizhen Lin, Mingwang Kong, Xiaowei Yao, Mingzhu Yin, Hui Xia, Jun Ma, Hongtao Liu

**Affiliations:** 1 Hubei University of Chinese Medicine, Wuhan, Hubei China; 2 Hubei Provincial Hospital of Traditional Chinese Medicine, Wuhan, Hubei China

**Keywords:** Intestinal microbiome, Non-alcoholic fatty liver disease (NAFLD), Gut–liver axis, Bile acids, Farnesoid X receptor

## Abstract

Non-alcoholic fatty liver disease (NAFLD) is the hepatic manifestation of dysregulated lipid and glucose metabolism, which is often associated with obesity, dyslipidemia and insulin resistance. In view of the high morbidity and health risks of NAFLD, the lack of effective cure has drawn great attention. In recent years, a line of evidence has suggested a close linkage between the intestine and liver diseases such as NAFLD. We summarized the composition and characteristics of intestinal microbes and reviewed molecular insights into the intestinal microbiome in development and progression of NAFLD. Intestinal microbes mainly include bacteria, archaea, viruses and fungi, and the crosstalk between non-bacterial intestinal microbes and human liver diseases should be paid more attention. Intestinal microbiota imbalance may not only increase the intestinal permeability to gut microbes but also lead to liver exposure to harmful substances that promote hepatic lipogenesis and fibrosis. Furthermore, we focused on reviewing the latest “gut–liver axis”-targeting treatment, including the application of antibiotics, probiotics, prebiotics, synbiotics, farnesoid X receptor agonists, bile acid sequestrants, gut-derived hormones, adsorbents and fecal microbiota transplantation for NAFLD. In this review, we also discussed the potential mechanisms of “gut–liver axis” manipulation and efficacy of these therapeutic strategies for NAFLD treatment.

## Introduction

Non-alcoholic fatty liver disease (NAFLD), the liver manifestation of metabolic syndrome, is a spectrum of liver disorders ranging from simple steatosis (non-alcoholic fatty liver) to non-alcoholic steatohepatitis (NASH) and even liver cirrhosis [1]. Recently, NAFLD has become the most common liver disease worldwide, and the global prevalence was estimated to be from 25 to 45% [2]. Noticeably, NASH is now the second leading etiology for liver transplantation, and a certain proportion of patients with NAFLD can potentially progress to hepatocellular carcinoma [3]. Since the progression of NAFLD is closely related to obesity and insulin resistance, the NAFLD incidence is expected to rise in parallel with the increased glucolipid metabolism disorder [4].

NAFLD is characterized by a diffused fat accumulation in vesicles that displace the cytoplasm of hepatocytes, i.e., steatosis. So far, the underlying mechanism behind the development and progression of NAFLD has not been fully elucidated. Historically, “two-hit” hypothesis was used to explain the pathogenesis of NAFLD [5]. However, this view was considered to be too simplistic to summarize the synergy of multiple stimulating factors in the occurrence of NAFLD. Currently, NAFLD is inclined to be a “multiple-hit” disease [6]. Such hits involve genetic, metabolic and environmental factors including epigenetic modifications, dietary intake, hormones (leptin, adiponectin) secreted from adipose tissue, crosstalk between different organs or tissues and so on [6]. Among these risk factors, a growing body of evidence indicates that gut–liver axis is implicated in the onset and progression of NAFLD. And the modification of gut–liver axis has been considered as a novel therapeutic approach for the management of NAFLD [7, 8].

This review summarized the role of intestinal microbiota in the occurrence of NAFLD. In addition, we assessed the therapeutic potential of intestinal microbiome manipulation for treating NAFLD and discussed the efficacy of these treatments.

## Intestinal microbiome

A variety of microbial communities are distributed on the surface of the human body as well as in the lumens of intestine, vagina and stomach [9]. The vast majority of these microbes live in our intestinal tract, which carries about 1.5 kg of symbiotic bacteria, above thousands of different species [10]. The human intestinal microbiota mainly includes bacteria, archaea, viruses and fungi [11]. Among these microbial groups, studies are mainly focused on bacteria, and the six dominant bacterial phyla of healthy adults are Firmicutes, Bacteroides, Proteobacteria, Actinobacteria, Fusobacteria and Verrucomicrobia [12]. Noticeably, nonbacterial microbes are also important for human health such as archaeal, fungal and viral populations, the species and quantities of which interact with each other [11]. Beyond bacteria, the non-bacterial intestinal microorganisms have been proved to be closely related to human diseases. For instance, fungi might play a role in the pathogenesis of inflammatory bowel disease (IBD) [13]. Thus, the crosstalk between non-bacterial gut microbes and human diseases should be paid more attention in the future.

**Fig. 1:**
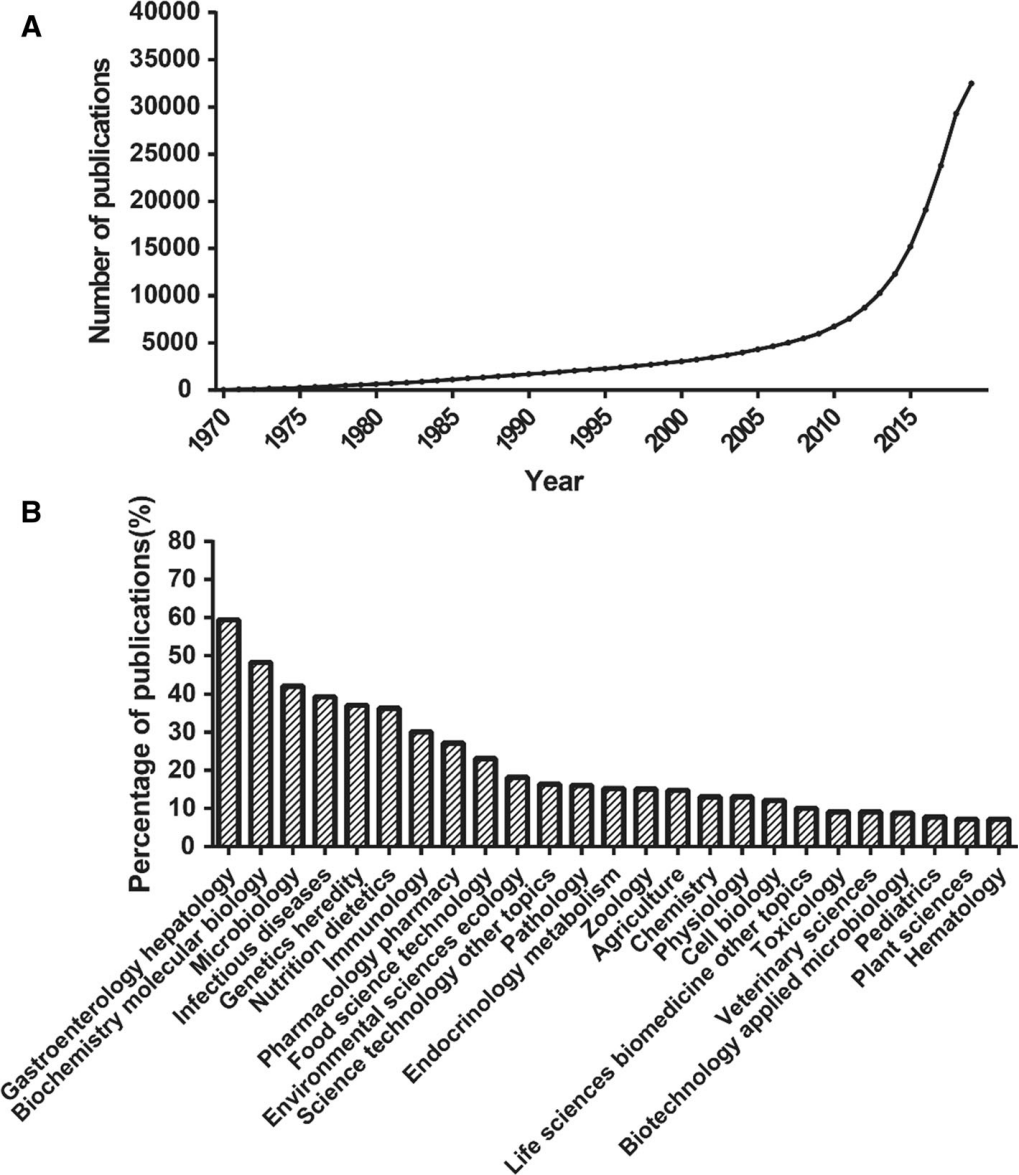
Statistical analysis of published articles about intestinal microbiome on Web of Science search engine (referencing the terms “intestinal microbiome”, “gut microbiota”, “intestinal flora”, “gut microorganisms” or “gut microbes”). **a** Annual publication of intestinal microbiome within the past 50 years. **b** Distribution of research fields based on the intestinal microbiome-related papers from 2010 to 2019

The intestinal microbiota provides various benefits for host health, including the maintenance of mucosal barrier integrity, bile acid metabolism, nutrient acquisition and prevention from the invasion of pathogens [14, 15]. For example, the gut bacteria express carbohydrate-active enzymes, which enable them to ferment unabsorbed and non-digestible carbohydrates producing metabolites such as short-chain fatty acids (SCFAs) [16]. These SCFAs, absorbed by intestinal epithelial cells, are involved in the regulation of inflammation, cell proliferation and mucus secretion [17]. With the gradual revealing of types and functions of gut microbes, their important roles in human health were proved by increasing studies. Since 2009, a high attention from researchers has been paid to intestinal microbiome, accompanied by the blowout of published articles (Fig. 1a). Since the intestinal microbiome populations are dynamic, their composition and distribution will change under different nutritional, immune and environmental conditions. The imbalanced intestinal flora may have profound impact on the physiological activities of host and even lead to the occurrence of a series of human diseases [14, 18].

## Gut–liver axis

The intestinal barrier is a complex system consisting of gut microbiota, intestinal mucosa, epithelial cell layer and blood vessels. This barrier is critical to retard harmful substances (toxins, microbes and bacterial metabolites) and maintain the normal environment of intestinal tract [19]. Under pathological conditions, the stimuli (e.g., toxins or intestinal inflammation) can change barrier permeability. And the impaired intestinal barrier will fail to prevent translocation of intestinal microorganisms and/or their products (also called pathogen-associated molecular patterns, PAMPs) into the mesenteric portal blood flow [20]. The precise mechanism by which gut microbes interact with intestinal barrier remains unclear. In addition to intestinal epithelial barrier (IEB), Spadoni and colleagues proposed a second intestinal barrier (gut-vascular barrier, GVB) below IEB in mice and humans [21]. The GVB, composed of endothelial cells, enteric glial cells and pericytes, prevents intestinal microbes from entering the body circulation [21, 22]. Further, Mouries et al. demonstrated that high-fat diet (HFD) induced dysbiosis that in turn disrupted GVB and drove the translocation of bacteria or their products into liver [23]. GVB-related research not only helps to understand the interaction between gut microbiota and intestinal barrier but also provides new insights into the prevention of NAFLD by regulation of gut–liver axis.

About 70% of the blood supply to liver comes from gut through the portal vein. The blood circulation enables liver to interact with intestinal bacterial products such as bacterial DNA, lipopolysaccharide (LPS) or intact bacteria due to the increased intestinal barrier permeability [24, 25]. Some of the translocated bacterial products can induce liver inflammation by binding to the specific pathogen recognition receptors (e.g., Toll-like receptors, TLRs) and promote the progression of liver disease [26]. Therefore, intestinal microbiome may be a crucial actor in the maintenance of gut–liver axis homeostasis and in the pathogenesis of liver diseases.

Based on statistical analysis of published articles in the past ten years, the studies on intestinal microbiome were mainly focused on gastroenterology hepatology, biochemistry molecular biology and other related life science fields. Of note, the investigations about gastroenterology hepatology ranked first, indicating the close relationship of intestinal microbiome to liver diseases such as NAFLD (Fig. 1b).

## Intestinal microbiota dysbiosis and NAFLD

**Fig. 2:**
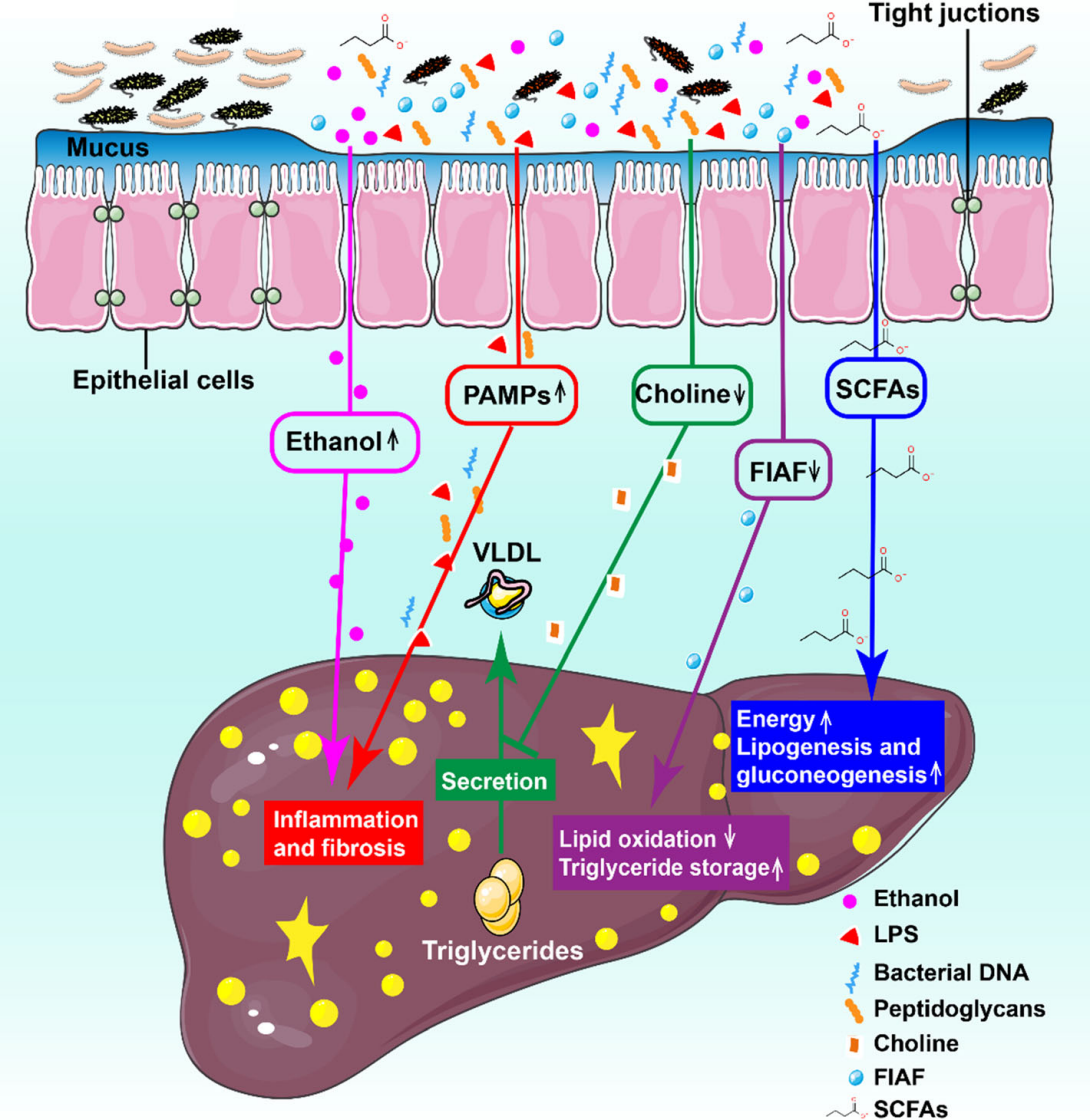
Schematic summary of intestinal microbiota dysbiosis responsible for the pathogenesis of NAFLD. The role of gut microbiota in occurrence of NAFLD is as followed: (1) microbial dysbiosis leads to the increased production of intestinal ethanol, which is toxic to liver and can damage the gut permeability by destroying tight junctions; (2) gut-derived pathogen-associated molecular patterns (PAMPs) such as LPS can bind to specific TLRs in liver and thus activates the proinflammatory pathways which result in hepatic inflammation and fibrosis; (3) gut microbiota hydrolyze choline to form dimethylamine and trimethylamine. Increased choline metabolism may cause choline deficiency, which prevents the excretion of very low-density lipoprotein (VLDL) and initiates the accumulation of triglycerides in liver; (4) an altered gut microbiota might inhibit the secretion of fasting-induced adipocyte factor (FIAF, also known as angiopoietin-related protein 4, ANGPTL4), a specific inhibitor of endothelial lipoprotein lipase (LPL), which releases triglycerides from VLDL particles into the liver. The net effects are inhibition of lipid β-oxidation and increased storage of hepatic triglyceride; (5) excessive short-chain fatty acids (SCFAs), substrates for gluconeogenesis and fat synthesis in liver, promote the accumulation of hepatic free fatty acids (FFAs) by inhibiting the activity of adenosine monophosphate activated protein kinase (AMPK)

Intestinal microbiota dysbiosis is defined as the loss of fragile equilibrium within various microbial entities in intestinal ecosystem [27]. Multiple researches show that the pathogenesis of human NAFLD is closely associated with the imbalance of intestinal microflora (Fig. 2) [11, 28]. So far, there have been a series of studies on the relationship between intestinal flora and NAFLD based on animal models or clinical trials. Next, we will give a brief summary of the role of intestinal microbiota dysbiosis in occurrence of NAFLD.

### Animal model studies

A number of studies have shown that the intestinal microbiota is a new environmental factor leading to obesity and NAFLD. First, Bäckhed and colleagues reported that the germ-free (GF) mice are protected against obesity produced by a Western-style, high-fat and high-sugar diet [29]. Moreover, the conventionalization of GF mice with a normal intestinal flora from conventionally raised animals increased the body fat content and insulin resistance within 14 days [30].

A direct involvement of gut microbes in the development of NAFLD was suggested by the finding that NAFLD could be delivered to GF mice by fecal microbiota transplantation (FMT) [31]. In this study, C57BL/6J mice fed with HFD usually showed hepatic steatosis and systemic inflammation (responders), but there were some other mice which were non-responders and failed to exhibit the symptom of metabolic disorders after HFD treatment. To explore the potential reasons for these inconsistent responses, the mouse gut microbes from responders or non-responders were transplanted to GF mice. Compared with the non-responder receiver group, the responder receiver group had a significant increase in fasting insulinemia and plasma aspartate aminotransferase (AST). Also, the responder receiver mice accumulated more hepatic triglycerides and subsequent liver steatosis [31].

Further studies suggest that the harmful metabolites produced by altered intestinal microbiome should be responsible for the development of NAFLD [32]. For example, Yamada and co-workers have reported the relationship between saturated fatty acids generated by intestinal microbiota and pathogenesis of NASH [33]. The authors found that feeding with STHD-01, a new class of HFD, led to the development of NASH in mice, accompanied by the dysbiosis and alterations in luminal metabolic profiles. This study revealed that the accumulation of saturated fatty acids, transformed from unsaturated fatty acids by gut microbiota, elicited inflammation by activating the migratory macrophages in liver [33]. More recently, Yuan et al. reported that the high alcohol-producing *Klebsiella pneumoniae* (HiAlc *Kpn*) was detected in 61% of individuals with NAFLD in a Chinese cohort [34]. To investigate the relationship between HiAlc bacteria and fatty liver disease, they fed specific-pathogen-free (SPF) mice with HiAlc *Kpn*. Strikingly, HiAlc *Kpn* feeding induced the chronic hepatic steatosis. Furthermore, the transfer of a HiAlc-*Kpn*-strain-containing microbiota from NASH patients by FMT into mice resulted in the occurrence of NAFLD. However, after selective depletion of the HiAlc *Kpn* strain by phage, there were no significant NAFLD symptoms in recipient mice. These results illustrated the contribution of a high level of alcohol-producing gut bacterial strain to pathogenesis of NAFLD [34]. Overall, the gut microbiota and their detrimental metabolites (ethanol, saturated fatty acids, polyamines, hydrogen sulfide, and so on) likely drive the damage to liver. And more studies are required to discover which intestinal microbes and/or their metabolites can promote the initiation and progression of NAFLD.

### Clinical research

Excessive gut-derived endotoxin will induce the production of ROS in liver, while ROS can damage vulnerable hepatic cells and thus lead to the occurrence of NASH [35]. In 2001, Wigg et al. reported a higher prevalence of small intestinal bacterial overgrowth (SIBO) in NASH patients compared with healthy subjects [36]. In 2009, Miele et al. examined the incidence and potential mechanism of increased intestinal permeability in NAFLD patients [37]. They found that NAFLD patients had significantly increased intestinal permeability in comparison with healthy volunteers. And this abnormality was associated with an increased prevalence of SIBO. It was supported by Harte et al. who reported that a higher circulating level of endotoxin was detected in patients with NAFLD and NASH compared with healthy controls [38].

In addition to gut-derived endotoxin, multiple studies have been performed to examine the difference of gut bacterial compositions between healthy subjects and NAFLD patients. Boursier and co-workers investigated the association of disbalanced intestinal bacterial community with severe NAFLD lesions (i.e., NASH and fibrosis) [28]. Multivariate analysis shows that the enrichment of *Bacteroides* genus was independently associated with NASH, and the increased abundance in *Ruminococcus* was positively related to the deteriorated fibrosis [28]. In a pediatric study, Zhu et al. described the alteration of gut microbiomes in patients with NASH, suggesting that children with obesity or NAFLD featured higher abundances of *Prevotella* and *Bacteroidetes* as compared to healthy controls [39]. More recently, Loomba et al. provided a novel method based on gut microbiome for non-invasive detection of advanced fibrosis in NAFLD patients [40]. Given the association between specific microbiota and NASH, it is possible to develop a panel of gut microbiome-derived biomarkers to predict advanced fibrosis.

Taken together, the above studies support the view that intestinal microbiota dysbiosis is a key environmental factor contributing to the NAFLD development and its progression into NASH.

## Targeting the gut–liver axis to treat NAFLD

The gut microbiota can induce liver inflammation by providing toll-like receptor ligands (e.g., LPS, peptidoglycan, bacterial flagella and DNA), which promote down-stream signaling events and thus lead to the secretion of proinflammatory cytokines [41]. Accumulating evidences have demonstrated that targeting the gut–liver axis might be a new approach to prevent or treat NAFLD, including the application of antibiotics, pre-/pro-/synbiotics and farnesoid X receptor (FXR) agonists.

### Antibiotics application to NAFLD treatment

To diminish the effects of microbial components and their metabolites on host health, antibiotics are usually used to reduce the number of intestinal flora. There are two types of antibiotics: absorbable antibiotics and non-absorbable ones. The former can effectively pass through the intestinal barrier to achieve therapeutic serum concentration, while the latter remains mainly within the gut milieu. Starting in the 1950s, antibiotics such as rifaximin, metronidazole and neomycin had been reported to treat patients with cirrhosis and hepatic encephalopathy [42, 43]. Also, the combined use of antibiotics (neomycin and polymyxin B) was proved to prevent fructose-induced hepatic lipid accumulation by decreasing the translocation of gut toxins [44].

Besides the suppression against local or systemic infection, antibiotics have regulatory effects on intestinal microbiota and are of benefit to NAFLD. For example, the treatment with cidomycin orally was found to promote small intestine transit rate and reduce serum levels of alanine aminotransferase (ALT), AST and TNF-α in NASH mouse model, indicating a potential of cidomycin in alleviating the severity of NASH by intestinal microbiota modulation [45]. Rifaximin, a largely water-insoluble and nonabsorbable (< 0.4%) drug, has been shown to exert antimicrobial activity against enteric bacteria such as *Streptococcus*, *Bacteroides* and *Citrobacter* [46]. Gangarapu et al. have demonstrated that a short-term administration of rifaximin (1200 mg/day for 28 days) improved the clinical status of patients with NAFLD/NASH, which was associated with reduced serum transaminases and circulating endotoxins [47]. Abdel-Razik et al. reported that after rifaximin therapy (1100 mg/day for 6 months), patients with NASH showed an evidently reduced levels of proinflammatory cytokines, ALT and NAFLD-liver fat score [48]. However, in an open-label clinical trial, rifaximin administration (800 mg/day for 6 weeks) was not effective for humans with NASH. The inconsistency may be due to the small sample size, the relatively low treatment dose or short duration in clinical study.

Specific antibiotics can act positively on intestinal microbiota and provided a so-called ‘eubiotic’ effect by promoting the growth of beneficial gut bacteria (e.g., *Bifidobacteria* and *Lactobacilli*), and this property may represent a therapeutic advantage in particular clinical practices [49]. On the other hand, although short-term treatment with antibiotics significantly improved NALFD, the long-term use of antibiotics should be careful in consideration of the possible side effects [50]. For example, antibiotics affect not only the harmful bacteria but also the healthy ones due to their wide ranges of action. Additionally, the use of antibiotics in immunocompromised or critically ill patients should be carefully evaluated to reduce the risk of infective endocarditis and bacteremia [51]. Besides, antibiotics may select for antibiotic-resistant strains in the human gut [52]. Collectively, the depletion or alteration of intestinal microbiota by antibiotics seems to alleviate the severity of NAFLD. However, considering the risk of antibiotics (side effects, resistance, etc.,), their clinical use must be cautious in treatment of NAFLD.

### Probiotics application to NAFLD treatment

Probiotics are preparations or products containing viable, defined microorganisms in adequate amounts. Probiotics exert beneficial effects in host by altering the composition of microbial flora [53]. So far, the main commercialized probiotics in market include Lactobacilli, Streptococci, Bifidobacterial and Fungi [54]. Although most probiotics are from bacteria, the yeast strain such as *Saccharomyces boulardii* has also been proven to be an effective probiotic [55].

Probiotics as a type of attractive therapeutic agents have been applied for the treatment of human NAFLD (Table 1). For example, VSL#3 is a probiotic mixture used for NAFLD in both animal experiments and clinical studies. The VSL#3 product has 450 billion bacteria per bag, which is a mixture of eight different bacteria (*Bifidobacterium longum, Bifidobacterium infantis, Bifidobacterium breve, Lactobacillus acidophilus, Lactobacillus bulgaricus Lactobacillus plantarum, Lactobacillus casei* and *Streptococcus thermophilus*) [56]. In a randomized controlled trial, the 4-month supplement of VSL#3 has been demonstrated to improve fatty liver and body mass index (BMI) in obese children with NAFLD. And the increase of total and active form of glucagon-like peptide 1 (GLP-1) could be responsible for the beneficial effects of VSL#3 [57]. In another clinical study, VSL#3 treatment significantly decreased the plasma levels of malondialdehyde (MDA), S-nitrosothiols and 4-hydroxynonenal in adult NAFLD patients [56]. A double-blind, randomized clinical trial was conducted using a probiotic mixture (containing 200 million of seven bacteria strains such as *Lactobacillus rhamnosus*, *Lactobacillus acidophilus*, *Streptococcus thermophilus* and *Bifidobacterium breve*) for 28 weeks on patients with NAFLD [58]. In this study, the probiotic consumption resulted in significant reductions in fibrosis score, hepatic inflammation and liver aminotransferases [58]. The result was further proved by Aller et al. who reported that the application of *Lactobacillus bulgaricus* and *Streptococcus thermophilus* significantly reduced the blood levels of ALT, AST and γ-glutamyltransferase (γ-GT) in patients with NAFLD, indicating the improved liver function [59].

**Table 1:** Clinical trials using antibiotics, probiotics, prebiotics, and synbiotics in NAFLD/NASH

Intervention	Agent	Trial phase	Target population	Results	Clinical Trials ID	References
Antibiotic	Rifaximin	–	NAFLD, *n* = 42	Reduction in serum AST, ALT, and endotoxin	NCT02009592	[47]
Antibiotic	Rifaximin	–	NASH, *n* = 50	Improved insulin resistance, cytokines, and NAFLD-liver fat score	NCT02884037	[48]
Antibiotic	Rifaximin	Phase 4	NASH, *n* = 15	No results posted	NCT01355575	–
Antibiotic	Solithromycin	Phase 2	NASH, *n* = 10	No results posted	NCT02510599	–
Probiotic	*Lactobacillus bulgaricus and Streptococcus Thermophilus*	–	NAFLD, *n* = 30	Reduction in ALT, AST	–	[59]
Probiotic	VSL#3	–	obese children with NAFLD, *n* = 48	Reduce fatty liver, BMI, GLP-1	NCT01650025	[57]
Probiotic	VSL#3	Phase 2	NAFLD, *n* = 20	No results posted	NCT03511365	–
Probiotic	Lactobacillus reuteri (L. reuteri) V3401	Phase 2	Obese subjects with insulin resistance, *n* = 60	No results posted	NCT02972567	[144]
Prebiotic	Oligofructose	Not applicable	NASH, *n* = 14	Reduced histologically-confirmed steatosis	NCT03184376	[82]
Prebiotic	Oat bran	Not applicable	NASH, *n* = 84	No results posted	NCT03897218	–
Prebiotic	Oligofructose-enriched inulin	Not applicable	NAFLD, *n* = 60	No results posted	NCT02568605	[145]
Prebiotic	Inulin/OFS 75/25	Not applicable	NAFLD, *n* = 60	No results posted	NCT02642172	–
Synbiotic	Fructo-oligosaccharide + 7 strains of bacteria	Phase 3	NASH, *n* = 42	Reduction in serum cytokines, hepatic steatosis and fibrosis	NCT02530138	[85]
Synbiotic	Lepicol probiotic and prebiotic formula	Not applicable	NAFLD, *n* = 20	Reduction in liver fat and AST level	NCT00870012	[146]
Synbiotic	Fructo-oligosachharide + Bifidobacterium animalis subsp. lactis BB-12	Not applicable	NAFLD, *n* = 104	No results posted	NCT01680640	[147]
Synbiotic	Fructooligosaccharide + 7 strains of bacteria	Phase 3	NAFLD, *n* = 52	Attenuation of inflammatory markers	NCT01791959	[58]

*No results posted* no results have been submitted to ClinicalTrials.gov., *ALT* alanine aminotransferase, *AST* aspartate aminotransferase, *TLR-4* Toll-like receptor 4, *BMI* body mass index

Probiotic treatment can protect the gut barrier from being damaged. The work by Karczewski et al. demonstrated that administration with *L. plantarum* strain WCFS1 enhanced the expression of Zonula occludens-1 (ZO-1) and Occludin close to the tight-junction structures [60]. MIYAIRI 588, a specific phenotype of the strain *C. butyricum*, has been used as a probiotic for treating colitis and antimicrobial-associated diarrhea [61, 62]. A recent study suggested that MIYAIRI 588 prevented the progression of steatosis to liver carcinogenesis in a rat NAFLD model [63]. Parallel studies from another group confirmed the same result, in which MIYAIRI 588 improved HFD-induced fatty liver in rats [64].

Collectively, these studies indicate that probiotics play a therapeutic role in NAFLD treatment. It seems that different probiotics may act on different target organs by changing the composition of intestinal microflora, producing antimicrobial peptides, reducing intestinal permeability or preventing the translocation of bacterial products [53]. Due to the multiple pathologic mechanisms of NAFLD, the combined administration of several probiotic strains may be more effective than a single one [65, 66]. Additionally, there are still some questions to be elucidated for the role of probiotics against NAFLD. For example, it is not clear how probiotics act on their specific target organs, and studies are required to clarify the crosstalk between probiotics and original bacterial inhabitants in the gut.

Noticeably, though probiotics have been recognized as a potential therapeutic tool for NAFLD, their beneficial effect should be further demonstrated by large randomized and controlled studies [67]. Until now, most of the studies that demonstrate the therapeutic effects of probiotics were mainly from Middle East countries. It is well known that the composition of gut microbiota is highly heterogeneous among the populations from different regions. Hence, the beneficial effects of probiotics on NAFLD/NASH need to be verified among people with ethnic difference, and the optimal formulations and dosages are also required to be determined in the development of commercialized probiotic products.

### Application of prebiotics and synbiotics to NAFLD treatment

Prebiotics, which contain no living microorganisms, are nondigestible food ingredients that can selectively promote the proliferation and/or activity of one or several gut microbes [68]. Synbiotics are combination of prebiotics and probiotics [69]. It is estimated that the average intake of prebiotic fiber is 1–4 g/d in United States and 3–11 g/days in Europe, which is related to the dietary habits of local residents [70]. In animal models, prebiotics are usually used at doses of 5–20% by weight.

There are numerous in vitro and clinical studies demonstrating that prebiotics and synbiotics can be used to treat NAFLD (Table 1). Common prebiotics include oligofructose (OFS), lactulose, inulin and Synergy1^®^ [71]. The de novo lipogenesis pathway was found to be threefold higher in patients with NAFLD, indicating that the increased de novo lipogenesis is a key feature of this disease [72]. Prebiotic supplementation may improve NAFLD by reducing the fatty acid synthesis pathway as has been shown in animal experiments [71, 73]. Kok et al. reported that feeding with 10% of oligofructose, a nondigestible but fermentable oligomer of β-d-fructose, significantly alleviated fructose-induced hepatic triglyceride (TG) accumulation in rats [74]. The decreased lipogenic capacity was due to the reduced gene expression of enzymes which regulated hepatic lipogenesis, such as acetyl co-A carboxylase and fatty acid synthase (FAS) [75, 76]. Cani et al. proved that dietary supplementation with oligofructose improved body weight gain, reduced adipose development, and controlled HFD-induced inflammation [77]. The study also suggests that oligofructose might control the occurrence of metabolic diseases by modifying gut microbiota in favor of *Bifidobacterial*, which has been shown to improve mucosal barrier function and reduce the level of gut endotoxin [77–79]. In recent years, our group focused on the improvement of prebiotic supplement on metabolic syndrome in obese or HFD-fed mice [80, 81]. The results indicate that chitosan oligosaccharides (COS), oligomers of β-(1–4)-linked d-glucosamine, displayed dramatically suppressive effect on glucolipid metabolism disorder, including alleviation of insulin intolerance, and prevention of intestinal barrier damage [80]. COS treatment also reshaped the unbalanced gut microbiota in NAFLD mice by upregulating populations of *Lachnospiraceae_UCG_001* and *Akkermansia*, and reducing abundances of *Lachnospiraceae NK4A136* group, *Alistipes*, *Helicobacter* and *Odoribacter* [80]. In parallel studies, treatment with N-acetylated chitooligosaccharides (NACOS) prevented the occurrence of NAFLD in HFD-fed mice by suppressing hepatic inflammation and lipid accumulation and promoting the growth of beneficial gut bacteria [81].

Other preclinical and clinical trials to test the potential benefits of prebiotics on NAFLD patients are underway (Table 1). More recently, in a pilot clinical trial, 14 NASH patients were allocated to oligofructose or placebo intervention for 9 months [82]. Despite no changes in body mass index (BMI), oligofructose treatment significantly improved hepatic steatosis and NAS score. In addition, oligofructose supplementation increased the abundance of *Bifidobacterium* spp, which was inversely associated with obesity and plasma LPS [83, 84].

In the previous study, Eslamparast et al. found that the synbiotic plus lifestyle modification was superior to lifestyle modification alone for NAFLD treatment [58]. Similar result was reported by Mofidi et al. who provided evidence that synbiotic supplementation improved the main features of NAFLD in patients with normal and low BMI through reduction in inflammatory indices [85]. In both clinical trials, patients were assigned to consume the same synbiotic capsules, containing seven strains of bacteria and fructo-oligosaccharide. Although the data from trials have shown that synbiotics may alter the progression of NAFLD (Table 1), the exact mechanism of their therapeutic effects remains to be determined, and the future work should focus on the elucidation of host energy balance, regulators of metabolism, as well as reshaping of gut microbiota in NAFLD.

Based on the results from animal experiments and clinical trials, both prebiotics and synbiotics have been recognized as a potential therapeutic tool for NAFLD. However, given the limited investigation in this field, the generalization of prebiotics/synbiotics for treatment of NAFLD needs to be confirmed by high-quality clinical trials. Additionally, though prebiotics are constituents of natural foods, there were still studies showing that the consumption of prebiotics in excess of 30 g/days would cause adverse gastrointestinal effects such as flatulence [86]. In considering the vast differences in intestinal microbiota, dietary habits and host health status, the doses of therapeutic prebiotics/synbiotics should be individually customized in clinical application.

### Targeting bile acid-related signaling pathways for NAFLD treatment

**Fig. 3:**
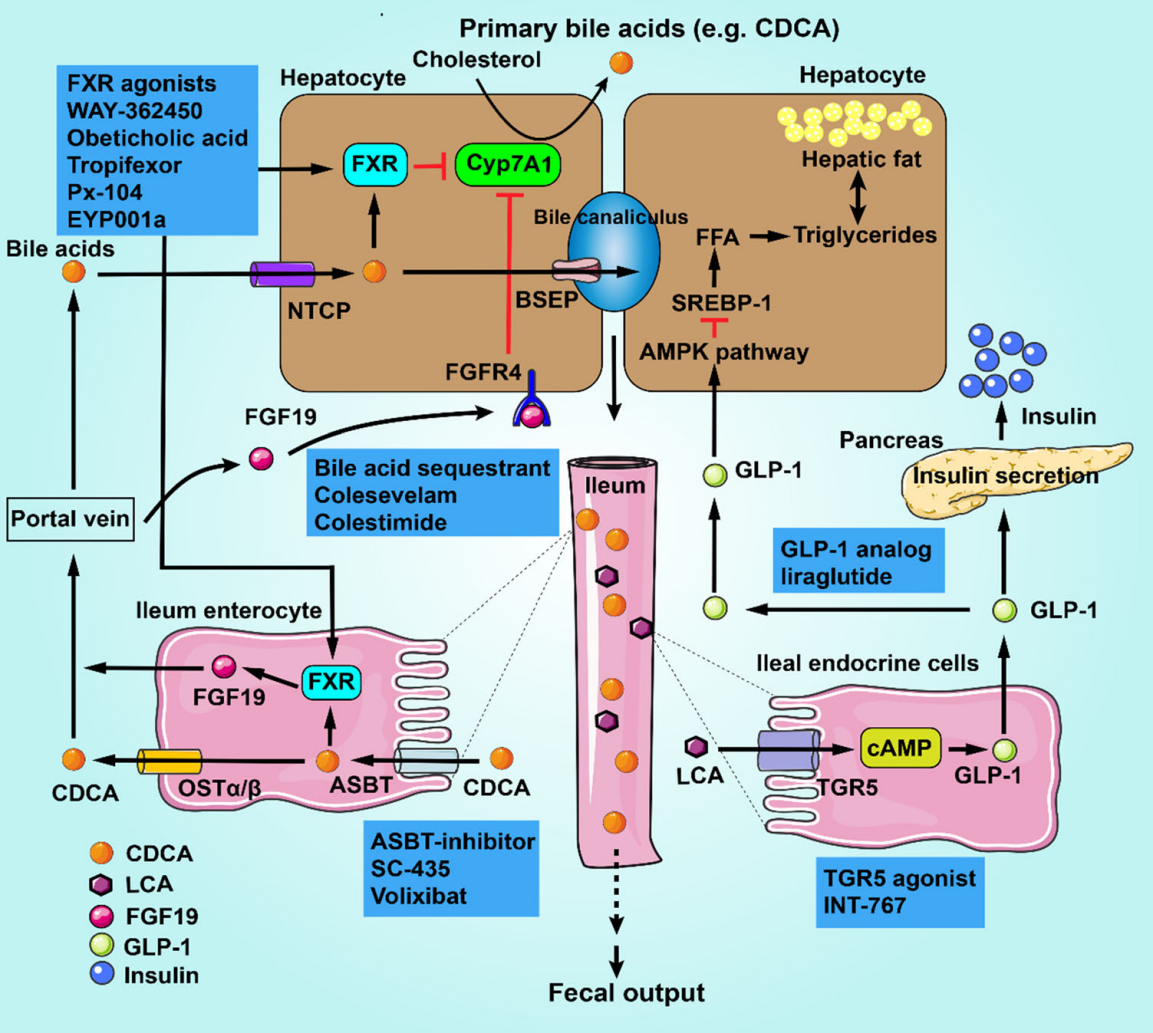
Schematic summary of bile acid (BA) biosynthesis, transport and metabolism. BAs are synthesized in hepatocytes via cytochrome P450 (CYP)-mediated oxidation of cholesterol to the primary bile acids through “classical” and “alternative” pathways, in which cholesterol 7-α-monooxygenase (CYP7A1) and sterol 27-hydroxylase (CYP27A1) are the major limited enzymes, respectively. BAs are transported into the bile canaliculus by bile salt export pump (BSEP). In ileum, bile salts are reabsorbed via apical sodium-dependent bile salt transporter (ASBT) in terminal ileum enterocytes. Activation of farnesoid X receptor (FXR) by bile salts releases fibroblast growth factor 19 (FGF19) into the portal circulation. FGF19 binds to its receptor fibroblast growth factor receptor 4 (FGFR4), and inhibits CYP7A1, thus repressing bile acid synthesis in hepatocytes. BAs in ileum enterocytes can also be secreted into the portal vein by organic solute transporter α/β (OST α/β), and then transported to hepatocytes via Na^+^-taurocholate cotransporting polypeptide (NTCP). In liver, BAs from the portal circulation bind to FXR, which activates small heterodimer partner (SHP) to repress CYP7A1. Primary bile acids (CA, CDCA) from the host are ligands for FXR, while secondary bile acids (LCA, DCA) from the microbiota are preferential ligands for Takeda-G-protein-receptor 5 (TGR5, also known as GPBAR1). In the ileal endocrine cells (L cells), activation of TGR5 stimulates the release of glucagon-like peptide-1 (GLP-1), which induces insulin secretion from the pancreas, and suppresses appetite and slows down gastric emptying. In addition, GLP-1 can inhibit liver fat accumulation via the cAMP/AMPK signaling pathway. *CA* cholic acid, *CDCA* chenodeoxycholic acid, *LCA* lithocholic acids, *DCA* deoxycholic acid, *FFA* free fatty acid, *SREBP-1* sterol regulatory element-binding protein 1

Bile acids are synthesized in hepatocytes via the oxidation of cholesterol and further converted to secondary bile acids by gut microbes [87]. After the transformation, about 95% of intestinal bile acids are reabsorbed across apical brush border membrane mediated by the apical sodium-dependent bile acid transporter (ASBT) in terminal ileum and then transported to liver via the portal vein, where they are absorbed by hepatocytes and resecreted into the bile [88]. In the intestinal tract, bile acids bind to receptors such as farnesoid X receptor (FXR) and Takeda G-protein-coupled receptor 5 (TGR5) to activate bile acid-related signaling pathways (Fig. 3) [89, 90], which will be involved in the development and progression of NAFLD/NASH [8]. And the exact mechanism is related to microbial enzymes, which are secreted by intestinal microorganisms and have various activities such as deconjugation, dehydroxylation and oxidation [91]. Emerging evidences suggest that gut microbes can regulate the pool size and composition of bile acids. For example, a small populations of *Clostridium* at genus level, including *C. hiranonis*, *C. sordelli* and *C. hylemonae*, are capable of producing secondary bile acids [92]; Bacteroides are the predominant intestinal bacteria responsible for conversion of CDCA to LCA by 7α-dehydroxylase activity [93]. On the other hand, the composition of gut microbiota can be altered by bile acids, FXR agonists and inhibitors against the bile acid absorption. It was demonstrated that cholic acid intake promoted the growth of several intestinal bacteria such as *Clostridia* and *Erysipelotrichi* in a rat model [94]. In a recent study, Pathak et al. investigated the effect of fexaramine (an intestine-restricted FXR agonist) on gut microbiome, hepatic glucose and insulin sensitivity. This study revealed that fexaramine treatment increased the abundances of *Acetatifactor*, *Bacteroides*, *Shewanella*, *Alistipes*, *Helicobacter* and F*lavonifractor*, but suppressed the growth of *Barnesiella*, *Prevolella*, *Clostridium *sensu stricto, *Turicibacter*, unclassified Prevotellaceae, unclassified Desulfovibrionaceae and unclassified *Turicibacter* [95]. Further studies indicate that bile acids can change the composition of gut microbes through direct antimicrobial action or FXR-induced antimicrobial peptides such as cathelicidin [94, 96]. As a major regulator of bile acid homeostasis, the expression of FXR was significantly decreased in livers of obese mice and patients with NAFLD [97, 98]. Moreover, the mice with FXR gene knockout exhibited liver steatosis and hyperlipidemia, which could be ameliorated by activation or overexpression of FXR [99, 100]. Bile acids and their related signaling pathways, mainly through activation of FXR and TGR5, play the key role in improving glucose/lipid metabolism as well as intestinal barrier function [8, 101].

Considering the effect of FXR and TGR5 signaling on glucose and lipid metabolism, it is believable that the molecules which can modulate both receptors (i.e., FXR agonists) or regulate endogenous levels of bile acids might have therapeutic effects on NAFLD/NASH (summarized in Fig. 3).

#### FXR agonists

It has been proved that FXR activation reduced the synthesis of bile acids and promoted their conjugation, transportation and efflux, thus protected liver from adverse effects triggered by excessive bile accumulation [102, 103]. So far, the frequently used FXR agonists are mainly as follows: natural FXR ligands (ursodeoxycholic acid, UDCA), nonsteroidal molecules (WAY-362450, PX-102 and GW4064) and derivatives of bile acids (obeticholic acid) [104].

UDCA, a naturally occurring hydrophilic bile acid, has been used to treat cholestatic liver diseases [105]. Recently, UDCA is considered as a potential therapeutic agent for NAFLD, and a small pilot trial shows that UDCA treatment significantly improved the liver enzymes and hepatic steatosis in NASH patients, but large‐scale studies suggest that UDCA was not effective for patients with NASH [106–108]. Therefore, the efficacy of UDCA for NAFLD/NASH treatment is required to be further confirmed.

WAY-362450 (also known as XL335 or FXR-450) developed by Flatt et al. is a highly potent and selective FXR agonist [109]. In the study using a mouse NASH model, WAY-362450 administration for 4 weeks significantly attenuated the inflammation and fibrosis in liver and reduced the levels of serum ALT and AST [110].

Px-102 (also known as Px20606), a synthetic FXR agonist, could potently lower the serum cholesterol level and significantly reduce the size of atherosclerotic plaques in animal models [111]. Oral treatment with Px-102 also reduced liver fibrosis development. Moreover, Px-102 reduced intestinal inflammation and bacterial migration from gut [112]. GW4064 is another selective nonsteroidal FXR agonist. As early as in 2006, Zhang et al. demonstrated that treatment with GW4064 significantly alleviated hyperglycemia and hyperlipidemia in diabetic db/db mice [99]. The FXR activation by GW4064 also alleviated diet-induced obesity and suppressed hepatic steatosis and insulin resistance [113]. Moreover, Yao and colleagues confirmed that GW4064 attenuated hepatic inflammation in a murine NAFLD model by suppressing the levels of proinflammatory cytokines and decreasing macrophage infiltration [114]. Collectively, these findings suggest that FXR activation by GW4064 may be a potential therapeutic option for NAFLD patients.

Among the reported FXR agonists, Obeticholic acid (also known as INT-747) was the most investigated one. Obeticholic acid, a 6α-ethyl derivative of CDCA, is a clinical-stage FXR agonist [115]. Len et al. reported that the treatment of Obeticholic acid exerted protective effect on cholestatic liver injury of rats [116]. The effect of Obeticholic acid on hepatic steatosis was studied in a NAFLD rat model, and the result shows that Obeticholic acid effectively improved the insulin resistance and lipid abnormalities, leading to a robust decrease in liver fibrosis [100]. Until now, the clinical studies using Obeticholic acid are still under way. In 2013, Mudaliar et al. first reported that Obeticholic acid treatment led to improved insulin sensitivity, suppressed hepatic inflammation and reduced fibrosis in patients with NAFLD [117]. Recently, a placebo-controlled clinical trial has been conducted to evaluate the effect of Obeticholic acid in adults with NASH [118]. In this study, patients were randomly assigned to receive Obeticholic acid or placebo for 72 weeks. Compared with the placebo group, treatment with Obeticholic acid was associated with the improvement in histological features of NASH, including hepatic steatosis, inflammation and hepatocellular ballooning. However, there were also adverse events observed in this clinical study such as abnormal cholesterol metabolism and the occurrence of pruritus. Hence, further studies should be conducted to confirm the long-term safety by Obeticholic acid administration.

Besides Obeticholic acid, some other FXR agonists are being tested in ongoing clinical trials (Table 2). For instance, the non-bile acid FXR agonist Tropifexor (LJN452) is being investigated in a double-blind, phase II, clinical trial in NASH patients (ClinicalTrials.gov identifier: NCT02855164). In addition, another clinical trial is being conducted to test the safety, tolerability and pharmacodynamics of a synthetic FXR agonist EYP001a in patients with NASH and to evaluate the influence of EYP001a on bile acid metabolism (NCT03976687).

**Table 2:** Compounds under clinical investigation for NAFLD/NASH, targeting the bile acid-related pathways

Intervention	Agent	Trial phase	Target population	Results	Clinical Trials ID	References
FXR Agonists	Obeticholic acid	Phase 2	NAFLD, *n* = 64	Reduction in body weight, hepatic inflammation and fibrosis, improved insulin sensitivity	NCT00501592	[117]
FXR Agonists	Obeticholic acid	Phase 2	NASH, *n* = 283	Reduction in ALT, AST, and γ-glutamyl transpeptidase, improved histological features of NASH	NCT01265498	[118]
FXR Agonists	Tropifexor	Phase 2	NASH, *n* = 351	No results posted	NCT02855164	–
FXR Agonists	EYP001a	Phase 1	NASH, *n* = 12	No results posted	NCT03976687	–
FXR Agonists	Cilofexor	Phase 2	NASH, *n* = 395	No results posted	NCT03449446	–
FXR Agonists	Px-104	Phase 2	NAFLD, n = 12	No results posted	NCT01999101	–
Bile acid sequestrant	Colesevelam	Phase 2	NASH, *n* = 54	Reduced LDL cholesterol, increases liver fat slightly	NCT01066364	[148]
Bile acid sequestrant	Colestimide	-	NASH, *n* = 38	A significant decrease of BMI, low-density lipoprotein cholesterol, and liver steatosis	–	[121]
ASBT inhibitor	Volixibat	Phase 2	NASH, *n* = 197	As none of the volixibat doses met the prespecified efficacy endpoints, the study was terminated	NCT02787304	-
ASBT inhibitor	Volixibat	Phase 1	Obese and overweight adults, *n* = 84	Increased bile acid synthesis, reductions in total cholesterol, and low-density lipoprotein cholesterol levels	NCT02287779	[149]

*No Results Posted* no results have been submitted to ClinicalTrials.gov

#### Inhibitors against bile acid absorption

Given that bile acid sequestrants have displayed improving effects on hepatic metabolism diseases, the blockade of intestinal bile acid absorption through sequestration represents a new strategy against NAFLD [8].

Colesevelam, a bile acid sequestrant, is a hydrophobic polymer with negligible absorption and systemic distribution. Colesevelam can block the enterohepatic circulation of bile acids and leads to increased conversion of cholesterol to bile acids in liver. In addition, Colesevelam treatment could reduce the plasma level of low-density lipoprotein cholesterol (LDL) and improve glycemic status in type 2 diabetic patients [119]. However, in a randomized and placebo-controlled clinical trial, Colesevelam treatment caused increased liver fat accumulation in patients with NASH while reduced LDL cholesterol [120]. Compared with Colesevelam, Colestimide (an anion-exchange resin) displayed a good therapeutic effect on patients with NASH and had no obvious side effects [121]. In the open-label trial, NASH patients treated with Colestimide (3 g/day) for 24 weeks showed significantly reduced levels of BMI and LDL cholesterol. Colestimide treatment was also associated with the significantly decreased visceral fat and ameliorated liver steatosis, which is the mainstay of NASH treatment.

Besides bile acid sequestrants, some ASBT inhibitors have been demonstrated to reduce the bile acid pool size and attenuate the hepatic inflammation and fibrosis [122]. In this respect, Anuradha et al. reported that the pharmacological blockade of ileal ASBT function using a luminally restricted inhibitor (SC-435) protected HFD-fed mice against NAFLD [123]. The authors observed that oral administration of SC-435 increased fecal bile acid excretion and suppressed mRNA levels of bile acid-responsive genes in ileum. Furthermore, SC-435 improved insulin sensitivity and prevented hepatic accumulation of triglyceride. More recently, a randomized placebo-controlled trial demonstrated that Volixibat, another ASBT inhibitor, increased the excretion of fecal bile acid and serum levels of 7α-hydroxy-4-cholesten-3-one (a bile acid synthesis biomarker) in healthy adults and patients with type 2 diabetes mellitus [124], further supporting the mechanistic rationale for application of ASBT inhibitors to NASH treatment. Generally, research about the effect of ASBT inhibitors on NAFLD are focused on the preclinically mechanistic studies. Thus, the well-controlled clinical trials are needed to determine the equivalence of these ASBT inhibitors between murine NAFLD/NASH models and human population.

### Role of intestinal hormones in the pathogenesis of NAFLD

Gastrointestinal tract is the largest endocrine organ in human body and secretes more than 20 different intestinal hormones [125]. Several G protein-coupled receptors (e.g., GPR41, TGR5) exist on the membrane of enteroendocrine L cells. The nutritional elements and some hormonal factors (insulin and leptin) can stimulate these receptors to induce the secretion of intestinal hormones such as ghrelin, Peptide YY and glucagon-like peptide 1/2 (GLP-1/2) [126, 127]. After being transported into systemic circulation, the intestinal hormones act on their target organs such as liver, adipose tissue and intestinal tract, thereby regulate NAFLD-related metabolic indices including glucose metabolism, insulin resistance and metabolic inflammation [128, 129]. Besides, intestinal hormones can be affected by the metabolites of gut microbiome, i.e., SCFAs [130]. A number of studies have suggested that intestinal hormones are critical to the pathogenesis of NAFLD and may become attractive targets for treatment of such disease [131, 132].

GLP-1, an incretin hormone secreted by L cells, can enhance glucose-induced insulin and inhibit the release of glucagon. The GLP-1 receptor agonist liraglutide has been recognized as a promising option for obesity treatment [133]. Wang et al. investigated the mechanism about the effect of liraglutide on weight control and reported that liraglutide could change the overall structure of intestinal microbiota in mice, leading to more lean-related phylotypes [134]. More recently, Moreira and co-workers investigated the effect of liraglutide on NAFLD in murine models of obesity [135]. It was found that liraglutide treatment led to obvious weight loss in obese mice, accompanied by the attenuated hepatic lipid accumulation. Moreover, microbiota analysis illustrated that liraglutide modified the diversity of gut microbiota by reducing the population of *Proteobacteria* and increasing the content of *Akkermansia muciniphila*, which were correlated with improved symptoms of NAFLD. Several clinical studies also demonstrated the efficacy and safety of liraglutide in patients with NASH or NAFLD [129, 136, 137]. Nevertheless, these clinical trials have not confirmed whether liraglutide exerts the efficacy by regulation of gut microbiota, and further investigations deserve to be performed to explore the potential relationship between therapeutic effect of liraglutide and its modulation on gut microbiota in patients with NAFLD or NASH.

### Application of adsorbent and fecal microbiota transplantation to NAFLD treatment

Endotoxaemia is also implicated in the pathogenesis of NAFLD [36]. By using adsorbents (a series of highly adsorptive materials), the toxins and bacterial products in gut were bound and thereby their flow into the liver and systemic circulation were suppressed [138, 139]. Since adsorbents fail to be adsorbed or degraded in gastrointestinal tract, they were mainly discharged in the way of stool. For example, AST-120 is a spherical carbon adsorbent (0.2–0.4 mm in diameter) with broad non-specific binding surface area (> 1600 m^2^/g), the application of which effectively lowered absorption of gut-derived ammonia into the body circulation in rats with chronic liver failure [140]. Recently, a synthetic activated carbon, i.e.,Yaq-001 (Yaqrit Ltd. UK), has been developed with optimized pore size in both macro- and micro-porous range [141]. Yaq-001 can selectively absorb intestinal-derived toxins such as cytokines, hydrophobic bile acid and bacterial products. Oral Yaq-001 therapy revealed a significant reduction in ALT and hepatic TLR-4 expression in rodents with NAFLD [142]. Currently, the clinical trials to assess therapeutic effect of Yaq-001 are under investigation as a part of the European Commission Horizon 2020 program (carbalive.eu) [141].

Fecal microbiota transplantation (FMT) is a new approach to clinical treatment, in which gut microbes are transferred from healthy donor to diseased recipient. By this way, a ‘healthy’ gastrointestinal microbiota may be reconstructed. Zhou and co-workers found that FMT intervention remarkably increased the concentration of butyrate in fecal contents and improved the tight junction of small intestine. This study further proved that FMT attenuated steatohepatitis in mice by a beneficial regulation of gut microbiota [143]. A growing interesting in FMT and its potential in liver diseases has been reflected in ongoing trials such as NAFLD/NASH (NCT03803540, NCT02469272 and NCT02721264) and liver cirrhosis (NCT02862249). In the future, further high-quality clinical data are needed to determine the efficacy and safety of FMT. And standardized protocols should be formed such as sample preparation, archiving, formulations and dosages.

## Perspectives and conclusions

NAFLD is a very common and severe disease which leads to cirrhosis and hepatocellular carcinoma and the prevalence of NAFLD/NASH is increasing worldwide. Intestinal microbiome mainly includes bacteria, archaea, fungi and viruses, and the association of non-bacterial gut microbes with human liver diseases should be paid more attention in the future. Recently, a series of studies have confirmed the critical role of intestinal microbiota in the maintenance of gut–liver axis balance and occurrence of NAFLD. Thereby, it is logical to target the gut–liver axis (especially the gut microbiota) to develop new strategies for NAFLD therapy. So far, high-quality preclinical researches and few randomized controlled trials have demonstrated the effectiveness of these therapies in NAFLD management. Considering that multiple therapeutic candidates based on gut–liver axis are still in the stages of in vitro or preclinical studies, more well-designed and mechanism-based laboratory and/or clinical investigations are required to confirm the efficacy of these medical agents for treatment of NAFLD.

## Abbreviations

ALT: Alanine aminotransferase
AST: Aspartate aminotransferase
BMI: Body mass index
FMT: Fecal microbiota transplantation
GLP-1: Glucagon-like peptide-1
NAFLD: Nonalcoholic fatty liver disease
NASH: Nonalcoholic steatohepatitis
PAMPs: Pathogen-associated molecular patterns
PYY: Peptide YY
ROS: Reactive oxygen species
SCFAs: Short chain fatty acids
SIBO: Small intestine bacterial overgrowth
TLRs: Toll-like receptors
LPS: Lipopolysaccharide
LDL: Low-density lipoprotein
